# Skeletal and dental effects on rats following *in utero*/lactational exposure to the non-dioxin-like polychlorinated biphenyl PCB 180

**DOI:** 10.1371/journal.pone.0185241

**Published:** 2017-09-28

**Authors:** Ashly N. Romero, Maria Herlin, Mikko Finnilä, Merja Korkalainen, Helen Håkansson, Matti Viluksela, Sabrina B. Sholts

**Affiliations:** 1 Department of Anthropology, University of Arkansas, Fayetteville, Arkansas, United States of America; 2 Institute of Environmental Medicine, Karolinska Institute, Stockholm, Sweden; 3 Research Group of Medical Imaging, Physics and Technology, University of Oulu, Oulu, Finland; 4 Department of Applied Physics, University of Eastern Finland, Kuopio, Finland; 5 National Institute for Health and Welfare, Chemicals and Health Unit, Kuopio, Finland; 6 Department of Environmental and Biological Sciences, University of Eastern Finland, Kuopio, Finland; 7 Department of Anthropology, National Museum of Natural History, Smithsonian Institution, Washington, District of Columbia, United States of America; University of Iowa, UNITED STATES

## Abstract

Polychlorinated biphenyls (PCBs) are a large class of persistent organic pollutants that are potentially harmful to human and wildlife health. Although a small number of dioxin-like (DL) PCBs are well characterized, the majority of PCBs have non-dioxin-like (NDL) modes of action and biological effects that are less understood. We conducted a dose-response study of the skeletal and dental effects of *in utero*/lactational exposure to 2,2',3,4,4',5,5'-heptachlorobiphenyl (PCB 180), a NDL PCB congener that is abundantly present in the environment and foods, including mother’s milk. In a sample of 35- and 84-day-old male and female offspring from pregnant rats exposed to different doses of PCB 180 (0, 10, 30, 100, 300, and 1000 mg/kg bw), we measured the three-dimensional (3D) coordinates of 27 landmarks on the craniofacial skeleton with a Microscribe G2X system, the buccolingual width of all molars with digital sliding calipers, and a variety of tibial parameters with peripheral quantitative computed tomography (pQCT) and a biomechanical testing apparatus. The landmark coordinates were analyzed for variation in size, shape, and fluctuating asymmetry (FA) using MorphoJ software, showing no effects on cranial size, on FA in females only (i.e., decreased asymmetry), and on shape in both sexes (i.e., decreased facial length and shift in the palatal suture). In the maxillary teeth, females in the highest dose group showed a significant decrease of 0.1 mm (*p* = 0.033) of the second molar only, whereas males in most dose groups showed average increases of 0.1 mm (*p* = 0.006–0.044) in all three molars. In the mandibular teeth, the only significant response to PCB 180 exposure was the average increase of 0.1 mm (p = 0.001–0.025) in the third molars of males only. Males also shower greater sensitivity in postcranial effects of increased tibial length and decreased cortical bone mass density, although only females showed significant effects on tibial bone area and thickness. These results demonstrate marked sex differences in effects of PCB 180, which can be attributed to differences in their underlying biological mechanisms of toxicity. Furthermore, although tooth and bone development are targets of both DL and NDL compounds, this study shows that there are marked differences in their mechanisms and effects.

## Introduction

Pollution is a major threat to human health and well-being in the 21^st^ century [1]. Although some environmental contaminants occur naturally on Earth, such as polycyclic aromatic hydrocarbons (PAHs) produced by volcanoes, forest fires, and petroleum seepage [2], many persistent organic pollutants (POPs) have been created by human activities within the last century. Until their production was banned in the 1970s, a large category of POPs known as polychlorinated biphenyls (PCBs) were manufactured and widely used for a variety of industrial applications [3]. For the same reasons that PCBs were effective coolants and lubricants for electrical equipment, e.g., low water solubility and high chemical stability, they are strongly resistant to chemical, biological, and photolytic degradation in the environment [4]. After decades of biogeochemical cycling, long-range transport, and bioaccumulation in food webs, variable concentrations of PCBs are routinely found in riverine fish [5, 6], marine mammals [7], and commercial fish, meat, and dairy products [8] in the European Union and the United States. As PCB exposure has been linked to a variety of adverse biological effects, including neurological dysfunction, reproductive and developmental problems, cancer, and impairments of the endocrine and immune systems [9, 10], the prevalence of these contaminants is a planetary health concern.

Most PCBs were manufactured as mixtures, with desired properties (e.g., viscosity) achieved by the "random" chlorination of biphenyl [11]. Given ten vacant positions on the biphenyl rings, PCBs have 209 possible substitution patterns, or congeners, defined and grouped by chlorine number and position. Congeners with no more than one chlorine substitution in the *ortho* position of the biphenyl ring(s) can assume a coplanar conformation, allowing them to bind with high affinity to the aryl hydrocarbon receptor (AHR) and elicit dioxin-like (DL) toxic effects. This group of 12 congeners, the DL-PCBs, is very small compared to the non-dioxin-like (NDL) PCB group, which consists of 197 *ortho*-rich (and thus non-coplanar) congeners that do not bind strongly to the AHR. However, even though NDL-PCBs are more numerous and abundant in the environment than DL-PCBs, less is understood about their toxic mechanisms and effects. Unlike DL-PCBs, for which the magnitude of toxic potency of individual compounds can be estimated as a toxic equivalency factor (TEF) in relation to 2,3,7,8-tetrachlorodibenzo-p-dioxin (TCDD), there is no such system in place to assess potential human or ecological risks due to NDL-PCB exposure [12]. Although the Joint FAO/WHO Expert Committee on Food Additives (JECFA) found that that dietary exposures to NDL-PCBs are unlikely to be of a health concern for adults and children, based on available data, it recommended that further toxicological studies should be done, particularly *in vivo* studies for congeners that contribute significantly to human dietary exposure and body burden [13].

We recently showed that TCDD exposure can alter cranial development and growth in rats, depending on the concentration, duration, and timing of the dose, as well as the sex and strain of the animal [14, 15]. Although these studies provide new insights into DL toxicity in the mammalian skull, the effects of NDL compounds on cranial formation remain unknown. To address this problem, we employed the same cranial morphometric methods used in these previous studies to analyze a sample of rats with *in utero* exposure to 2,2',3,4,4',5,5'-heptachlorobiphenyl (PCB 180). This NDL-PCB was selected for this study as it represents a large proportion of the NDL-PCB contamination of food and feed [10], as well as having a long elimination half-life of over 10 years [16, 17] and rapid rate of placental transfer within 2.5 hours [18]. Furthermore, PCB 180 is known to cause a unique toxicological profile, including sexually dimorphic skeletal effects, in a rodent 28-days repeat dose regulatory toxicology study [19]. To expand these observations, the present study was performed to cover cranial, dental, and tibial tissues. To address windows of development that might be particularly vulnerable to PCB exposure, we chose an *in utero*/lactational study design with a regulatory toxicological approach in terms of PCB180-dosing schemes and dose-response analyses of the observations. Laboratory rats were exposed directly to PCB 180 during pregnancy, and subsequent effects of *in utero* and lactational exposure on their offspring were measured on postnatal days (PND) 35 and 84. These two time points were selected (1) to study effects soon after cessation of the quantitatively significant lactational exposure (PND 35), (2) to monitor the possible further alterations towards adulthood without further exposure to PCB 180 (PND 84), and (3) to cover both young (PND 35) and adult (PND 84) animals. By detailed quantitative analyses of three-dimensional (3D) shape changes in craniofacial size and shape with geometric morphometrics (GM) techniques, combined with detailed cranial non-metric, odontometric, and postcranial measurements, we provide comprehensive dose-response data on skeletal development following NDL-PCB exposure during the early stages of life and its differential impacts on female and male rats into puberty and early adulthood.

## Materials and methods

### Chemicals

PCB 180 (2,2’,3’,4,4’,5,5’-heptachlorobiphenyl; CAS 35065-29-3; molecular weight 395.3; batch No. 6115) was purchased from Chiron, Trondheim, Norway and analyzed. The purity of PCB 180 as stated by the supplier was 98.9% and the analyzed level of dioxin-like impurities as represented by sum of WHO-TEQ contamination was 2.7 ng/g PCB 180. The PCB was dissolved in purity controlled (0.2 pg WHO-TEQ/g) corn oil (Sigma Aldrich, Munich, Germany; batch No. 065K0077), which served also as the vehicle for the unexposed (control) groups.

### Animals and treatment

The study protocol was approved by the National Animal Experiment Board of Finland (License No. ESLH-2006-07965/Ym23). Female and male Sprague Dawley rats were obtained from Harlan (Zeist, The Netherlands) and housed in single stainless steel cages (floor size 1380 cm^2^) at the animal house of THL (National Institute for Health and Welfare, Kuopio, Finland). Cages were supplied with a bedding of aspen chips (Tapvei Co., Kaavi, Finland). Rooms had a 12-h light: 12-h dark cycle, with the light phase starting at 7 am. The ambient temperature was 21°C ± 2°C, and relative humidity was maintained at 50% ± 2%. Standard laboratory rodent diet (R36, Lactamin, Sweden) and water were available ad libitum. For mating, one female was transferred to the cage of one male. The morning of the day on which females were sperm-positive was taken as gestational day 0 (GD 0). Thereafter, females were housed in single cages.

Pregnant females were randomly assigned to one of six dose groups (7 dams per group), which were exposed to PCB 180 dissolved in corn oil daily on GDs 7–10 by oral gavage. Total dose levels of PCB 180 were 0, 10, 30, 100, 300, or 1000 mg/kg body weight and the dosing volume was 4 ml/kg body weight. Controls received only the vehicle. Litters were standardized to 8 pups (4 males and 4 females) on PND 1. Pup body weights were measured on PND 1, PND 4, and, then, once a week, starting on PND 7. Offspring were weaned on PND 28. After weaning, littermates of the same sex were housed together in one cage. One offspring of each litter and sex was killed by CO_2_ asphyxiation and exsanguination on PND 35 and PND 84.

### Cranial measurements

Crania were preserved in 10% neutral buffered formalin, transferred to jars with soapy water, dissected, and cleaned prior to measurement. As the PND 35 offspring were too small for cranio-dental analysis by direct measurement, a total of 81 crania of PND 84 offspring were analyzed. Based on a previous study of TCDD exposure in rats, each cranium has 53 potential anatomical landmarks across three morphological components, i.e., the face, the vault, and the base [14]. Before measurement, all observable landmarks were marked in pencil on each cranium for clearer visibility and precision. Many landmarks were not present, due to previous brain extraction in the specimens that damaged the cranial vault and base. All landmarks on the face (n = 27) and several on the base (n = 4) and vault (n = 2) were observable on all specimens, resulting in the same dataset used in a recent study of TCDD exposure [15] (S1 Fig and S1 Table). The three-dimensional coordinates of each landmark were recorded with a MicroScribe G2X system (RevWare Inc.) by the same observer in two trials over two days. The presence or absence of one non-metric cranial trait, the interfrontal bone, was assessed visually and recorded simultaneously (S2 Fig).

### Dental measurements

After all cranial data were recorded, one odontometric distance (maximum buccolingual diameter) was measured on the right and left first (M^1^), second (M^2^), and third (M^3^) maxillary molars and first (M_1_), second (M_2_), and third (M_3_) mandibular molars using digital sliding calipers. Each tooth was measured three times by the same observer in three trials on the same day. The presence or absence of one non-metric dental trait, third molar agenesis, was assessed visually and recorded simultaneously.

### Tibial measurements

From all PND 35 and PND 84 offspring, the right tibia was defrosted, dissected and cleaned for measurement. The maximum length of each bone was measured once using digital sliding calipers by the same observer on the same day. A total of 82 right tibiae from PND 35 offspring and 81 right tibiae from PND 84 offspring were scanned using a peripheral quantitative computed tomography (pQCT) system (Stratec XCT Research SA+) with software version 5.50 (Norland Stratec Medizintechnik, GmbH, Birkenfeld, Germany). The voxel size was 0.07 mm. Single scans of the metaphysis and diaphysis of each tibia were performed, measured from the growth plate at 10% and 45% of the bone length, respectively. The thresholds for defining trabecular bone were 280 and 400 mg/cm^3^, and cortical bone was defined above a threshold of 710 mg/cm^3^. From these scans, measurements of bone mineral density (BMD), surface area, and thickness (for the cortical bone only) were obtained.

For biomechanical testing, tibial shafts were subjected to a three-point bending test using a universal testing machine (Instron 3366, Norwood, MA, USA) and a previously established protocol that was adapted for this study [20, 21]. Each bone was placed with its anterior surface upwards on a support with a span length of 13 mm, and bending load was applied with a constant speed of 0.155 mm/sec until failure. Bending stiffness was calculated as the slope of the linear part of load-displacement curve. Bone strength and toughness were measured as maximal force and absorbed energy to fracture, respectively.

### Geometric morphometrics

Because not all of the 53 cranial landmarks were observable on all specimens in the sample, only 27 landmarks were used in this study. Specimens without these 27 landmarks were excluded from the GM analysis, resulting in a subsample of 72 specimens, although all 81 specimens measured for cranial landmarks were amenable to analyses of the teeth, tibia, and interfrontal bone.

For the GM analysis, cranial landmark coordinates were scaled, rotated, and translated by a Procrustes fit in MorphoJ [22]. The coordinates from the two trials were averaged and subdivided by sex, and principle components analysis (PCA) was performed separately on female and male datasets. Shape variation within each dataset was partitioned into symmetric and asymmetric components by Procrustes superimposition of the original configurations and their mirror images. The symmetric component of shape variation was quantified as the averages of the original and reflected configurations, and the asymmetric component as their differences [23]. One measure of cranial size, centroid size (CS) was defined as the square root of the sum of squared distances between each landmark and the centroid of the landmark configuration for each individual.

### Statistical analysis

With the exception of the biomechanical variables, Shapiro-Wilk and Levene’s tests confirmed that the data met parametric assumptions of normality and equal variances with Stata version 10 software. For these data, unpaired *t-*tests were used to assess mean differences between the control and dose groups of female and male rats for different variables, and robust correlation tests were used to measure the linear association between pairs of variables with Pearson’s product moment correlation coefficient (*r*). For the biomechanical data, the Mann-Whitney U test was used as a non-parametric alternative to the *t*-test. Procrustes ANOVAs were used to test the effects of dose, individual variation, directional asymmetry (DA), fluctuating asymmetry (FA), and measurement trial on centroid size (CS) and shape in MorphoJ [22].

### Dose-response modeling

A family of exponential models (PROAST version 38.9 [RIVM, Bilthoven, The Netherlands] in the R software version 3.1.0, [R Development Core Team, R Foundation for Statistical Computing, Vienna, Austria]) was used for PCB 180 dose-response modeling of tooth size and tibial measurements. Based on the likelihood ratio test, the appropriate model was fitted to the data acquired from each strain. The benchmark doses (critical effect dose, CED) as well as CEDs at the upper and lower bounds of 95% confidence (CED-U and CED-L) were calculated at a 5% and 50% change in response compared with unexposed subjects [24].

## Results

### Bodyweight development and tissue concentration

Exposure to PCB180 induced slight and transient reductions in maternal bodyweight at the highest dose level (1000 mg/kg bw). Mean maternal bodyweights (±SD) on GD 20 (one day before delivery) were 383 ± 17, 382 ± 19, 382 ± 32, 380 ± 18, 384 ± 12 and 371 ± 35 g at 0, 10, 30, 100, 300, or 1000 mg/kg bw, respectively. Decreased bodyweights were also transiently observed in pups at the same dose level in the first postnatal week, but normalized thereafter, and there were no treatment related differences in bodyweights on PND 35 or 84 (Table 1). Neonatal mortality of offspring slightly increased at 300 and 1000 mg/kg.

**Table 1:** Measurements of tibial length (mean ± SD, mm), bone mineral density (mean ± SD, mg/cm^3)^, area and thickness (mean ± SD, mm) of diaphyseal cortical and metaphyseal trabecular bone by total dose of PCB 180 and sampling time point. Bodyweights at PND 35 and PND 84 (mean ± SD, g) are also shown.

Dose (mg/kg bw)		Tibia	Diaphysis, cortical			Metaphysis, trabecular		Body
	N	Length	BMD	Area	Thick.	BMD	Area	BW
		(mm)	(mg/cm^3^)	(mm^2^)	(mm)	(mg/cm^3^)	(mm^2^)	(g)
*Female*, *PND 35*								
0	7	27.7 ± 0.8	1159 ± 21	1.7 ± 0.1	0.3 ± 0.01	168 ± 36	6.0 ± 0.4	102 ± 13
10	7	28.3 ± 0.6	1164 ± 13	1.6 ± 0.1	0.3 ± 0.02	176 ± 25	6.0 ± 0.7	108 ± 10
30	7	27.9 ± 0.5	1160 ± 11	1.8 ± 0.3	0.3 ± 0.03	168 ± 48	5.8 ± 0.5	104 ± 10
100	7	27.9 ± 0.8	1149 ± 17	1.6 ± 0.1	0.3 ± 0.01	178 ± 13	5.8 ± 0.6	103 ± 11
300	7	28.5 ± 1.1	1155 ± 15	1.8 ± 0.2	0.3 ± 0.02	174 ± 31	6.1 ± 0.4	110 ± 11
1000	6	28.1 ± 0.7	1143 ± 22	1.8 ± 0.1	0.3 ± 0.01	155 ± 32	6.1 ± 0.6	99 ± 13
*Male*, *PND 35*								
0	7	28.0 ± 1.0	1143 ± 26	1.7 ± 0.3	0.3 ± 0.03	166 ± 32	5.1 ± 0.5	117 ± 18
10	7	28.6 ± 0.8	1142 ± 15	1.7 ± 0.2	0.3 ± 0.02	164 ± 40	5.4 ± 0.8	124 ± 15
30	7	28.7 ± 0.5	1149 ± 13	1.9 ± 0.2	0.3 ± 0.02	174 ± 16	5.4 ± 0.2	119 ± 11
100	7	28.7 ± 0.6	1144 ± 18	1.8 ± 0.2	0.3 ± 0.02	158 ± 18	5.3 ± 0.5	121 ± 14
300	7	28.9 ± 0.9	1144 ± 22	1.8 ± 0.3	0.3 ± 0.03	167 ± 30	5.5 ± 0.5	126 ± 15
1000	6	28.4 ± 0.8	1137 ± 19	1.8 ± 0.2	0.3 ± 0.02	167 ± 31	5.2 ± 0.4	108 ± 16
*Female*, *PND 84*								
0	7	37.0 ± 0.7	1331 ± 5	3.5 ± 0.2	0.5 ± 0.02	205 ± 26	5.2 ± 0.4	234 ± 21
10	7	37.5 ± 0.7	1333 ± 10	3.6 ± 0.1	0.5 ± 0.02	209 ± 15	4.9 ± 0.5	241 ± 14
30	5	37.3 ± 0.8	1332 ± 8	3.6 ± 0.2	0.5 ± 0.02	219 ± 38	**4.4 ± 0.3** ^**b**^	240 ± 17
100	7	37.4 ± 0.4	1335 ± 5	3.5 ± 0.1	0.5 ± 0.02	199 ± 48	4.8 ± 0.6	233 ± 15
300	7	**38.0 ± 0.5**^**b**^	1333 ± 13	**3.8 ± 0.2**^**a**^	**0.5 ± 0.02**^**a**^	217 ± 26	5.1 ± 0.5	247 ± 22
1000	5	36.8 ± 0.6	1325 ± 8	3.8 ± 0.1^a^	**0.6 ± 0.01**^**a**^	229 ± 29	**4.5 ± 0.5**^**a**^	240 ± 14
*Male*, *PND 84*								
0	7	39.4 ± 1.0	1326 ± 9	4.2 ± 0.5	0.6 ± 0.04	161 ± 15	7.5 ± 0.8	352 ± 39
10	7	**40.5 ± 0.8**^**a**^	1324 ± 8	4.5 ± 0.2	0.6 ± 0.02	170 ± 12	8.1 ± 0.6	377 ± 39
30	9	40.0 ± 1.0	1327 ± 11	4.3 ± 0.2	0.6 ± 0.02	167 ± 20	7.6 ± 0.6	350 ± 31
100	7	**40.5 ± 0.6**^**a**^	**1304 ± 8**^**c**^	4.4 ± 0.2	0.6 ± 0.02	163 ± 20	8.1 ± 0.6	381 ± 27
300	6	**41.4 ± 1.0**^**b**^	**1308 ± 9**^**b**^	4.5 ± 0.2	0.6 ± 0.02	166 ± 13	8.3 ± 0.4	395 ± 22
1000	7	39.6 ± 1.1	**1299 ± 11**^**c**^	4.3 ± 0.3	0.6 ± 0.02	176 ± 23	7.8 ± 0.4	352 ± 31

BMD = bone mineral density; BW = bodyweight; Thick. = thickness

Statistically significant mean differences from the control group are bold.

^a^ *p*<0.05

^b^ *p*<0.01

^c^ *p*<0.001 vs. control

Adipose tissue PCB 180 concentrations (to be published separately) in dams and in the offspring indicated lack of PCB 180 contamination of controls and reflected the administered total dose levels. In the offspring, pooled samples of controls, 100 and 1000 mg/kg bw were analyzed, and concentrations were slightly higher in females than in males. On PND 35 the concentrations at 1000 mg/kg bw were 9.6 (females) and 9.0 (males) times higher than at 100 mg/kg bw. On PND 84 adipose tissue PCB 180 concentrations at 100 mg/kg bw had decreased to 29.5% (females) and 24.6% (males) from those on PND 35. The same figures for 1000 mg/kg bw were 42.9% in females and 19.6% in males.

### Cranial morphometrics

Mean and standard deviation (SD) values for CS are reported by sex and dose in Table 2. Centroid size showed no effects of near significance with the exception of an average increase among males in the 100 mg/kg bw dose group (CS = 45.6±1.1, *p* = 0.055). Consistent with these results, only males showed significant effects of dose on CS (*p* = 0.019), accounting for 33.6% of total variation (Table 3). In contrast, only females in the two highest dose groups experienced a significant decrease in FA compared to the control group (*p* = 0.01–0.030) (Table 2).

**Table 2:** Centroid size (mean ± SD, mm) and fluctuating asymmetry (mean ± SD) for craniofacial landmarks in female and male offspring on PND 84 by total dose of PBC 180.

Dose (mg/kg bw)	N	CS (mm)	FA
Female			
0	5	41.5±1.4	0.024±0.003
10	7	41.8±0.7	0.022±0.004
30	5	41.9±1.2	0.021±0.004
100	5	41.9±0.8	0.021±0.003
300	7	42.2±0.7	0.019±0.003^a^
1000	5	41.6±1.2	0.019±0.002^a^
Male			
0	7	43.7±1.8	0.020±0.002
10	7	45.0±0.9	0.018±0.003
30	7	45.0±1.3	0.020±0.003
100	5	45.6±1.1	0.018±0.004
300	6	45.0±1.0	0.018±0.003
1000	6	43.2±1.5	0.021±0.002

CS = centroid size; FA = fluctuating asymmetry

Statistically significant mean differences from the control group are bold.

^a^ *p*<0.05 vs. control

**Table 3:** Results of ANOVA for the effects of dose of PCB 180, individual, and trial on centroid size for female and male offspring on PND 84.

	% SS	SS	MS	df	F	*p*
*Female*						
Dose	11.5	8.0	1.6	5	0.9	0.5229
Individual	77.8	54.0	1.9	29	8.7	< .0001
Error (trial)	10.8	7.5	0.2	35		
*Male*						
Dose	33.6	49.4	9.9	5	3.2	0.0185
Individual	64.6	94.9	3.1	31	41.3	< .0001
Error (trial)	1.9	2.7	0.1	37	-	-

SS = sums of squares; MS = mean squares; df = degrees of freedom

The ANOVA results for shape showed significant effects of dose and directional asymmetry (DA) in both males and females (*p*<0.001), but only males showed a significant effect of FA on shape (*p* = 0.0003) (Table 4). For both sexes, individual variation and measurement trial accounted for the majority of the total variation. As illustrated by the PCA results (S2–S7 Figs), the first and second PCs (accounting for 27% and 15% of total shape variation in females and 19% and 15% of total shape variation in males, respectively) did not clearly separate most dose groups. The control and highest dose groups (1000 mg/kg bw) show some separation in their second PC scores (S2 and S3 Figs), corresponding to shortening of the face in females (S5 Fig) and elongation of the face and palate in males (S7 Fig).

**Table 4:** Results of ANOVA for the effects of dose of PCB 180, individual, and trial on shape for female and male offspring on PND 84.

	% SS	SS	MS	df	F	*p*
*Female*						
Dose	11.7	0.03	0	195	2.6	< .0001
Individual	26.7	0.06	0	1131	2.4	< .0001
Side (DA)	17.4	0.04	0	35	51.4	< .0001
Ind * Side (FA)	11.5	0.03	0	1190	0.8	1.000
Error (trial)	32.6	0.07	0	2590	-	-
*Male*						
Dose	9.6	0.02	0	195	1.8	< .0001
Individual	33.3	0.06	0	1209	2.6	< .0001
Side (DA)	19.4	0.03	0	35	52.6	< .0001
Ind * Side (FA)	13.3	0.02	0	1260	1.2	0.0003
Error (trial)	24.5	0.04	0	2738	-	-

SS = sums of squares; MS = mean squares; df = degrees of freedom

### Cranial non-metrics

Interfrontal bones were not observed in either of the highest dose groups for either sex (100, 300, or 1000 mg/kg bw) (Table 5). The highest frequencies were 33% in the female control group (2/6), followed by 14% in the male control group and low dose groups of both sexes (1/7).

**Table 5:** Interfrontal bone frequency and maximum buccolingual diameter (mean ± SD, mm) for maxillary molar teeth in female and male offspring on PND 84 by total dose of PCB 180.

		M^1^		M^2^		M^3^	
Dose	IFB	Left	Right	Left	Right	Left	Right
(mg/kg bw)							
*Female*							
0	2/6	1.98±0.05	2.02±0.09	2.00±0.04	2.00±0.05	1.54±0.05	1.58±0.06
10	1/7	2.00±0.05	2.04±0.09	2.00±0.04	2.00±0.04	1.56±0.05	1.60±0.07
30	0/5	1.99±0.03	2.00±0.04	2.00±0.06	2.02±0.05	1.54±0.04	1.57±0.03
100	0/7	1.99±0.04	1.99±0.02	1.97±0.01	1.98±0.04	1.53±0.06	1.59±0.03
300	0/7	2.02±0.04	2.03±0.05	2.01±0.04	2.03±0.06	1.55±0.04	1.65±0.06
1000	0/5	1.97±0.04	1.97±0.05	**1.95±0.02**^**a**^	1.96±0.05	1.47±0.06	1.55±0.14
*Male*							
0	1/7	1.96±0.06	1.95±0.06	1.97±0.05	1.96±0.06	1.51±0.08	1.54±0.07
10	0/7	**2.01±0.09**^**a**^	2.01±0.09	2.02±0.06	**2.06±0.06**^**b**^	1.60±0.04	1.61±0.06
30	0/9	2.00±0.06	2.00±0.06	2.00±0.06	2.02±0.06	1.57±0.06	1.59±0.06
100	1/7	2.02±0.04	**2.03±0.05**^**a**^	2.02±0.04	**2.04±0.04**^**a**^	**1.59±0.03**^**a**^	**1.62±0.04**^**a**^
300	0/7	2.03±0.07	2.03±0.08	**2.05±0.06**^**a**^	**2.08±0.08**^**b**^	**1.61±0.04**^**a**^	**1.62±0.06**^**a**^
1000	0/7	1.98±0.09	1.99±0.08	1.97±0.10	2.03±0.07	1.49±0.07	1.54±0.10

IFB = interfrontal bone; M^1^ = first maxillary molar; M^2^ = second maxillary molar; M^3^ = third maxillary molar

Statistically significant mean differences from the control group are bold.

^a^ *p*<0.05

^b^ *p*<0.01 vs. control

### Odontometrics

Mean and SD values for tooth measurements are reported by sex and dose in Tables 5 and 6, and the outcome of dose-response modeling in Table 7A. All of the molars in both sexes were fully erupted, with the exception of one left M_3_ of a female in a low dose group (10 mg/kg bw). In the maxillary dentition, male tooth size exhibited greater sensitivity to PCB 180 compared to females, as all three molars on both sides showed size increases at low and moderate doses of PCB 180, i.e., 10–300 mg/kg bw, without dose-dependence (Table 5). It is noteworthy that at the highest dose (1000 mg/kg bw) the molar size did not differ from controls (*p* = 0.071–0.947). In females, the only significant effect was a size decrease in the M^3^ at the highest dose level (*p* = 0.001–0.0424).

**Table 6:** Third molar agenesis frequency and maximum buccolingual diameter (mean ± SD, mm) for mandibular molar teeth in female and males rats by total dose of PCB 180.

		M_1_		M_2_		M_3_	
Dose	3MA	Left	Right	Left	Right	Left	Right
(mg/kg bw)							
*Female*							
0	0/6	1.69±0.07	1.73±0.08	1.94±0.05	1.92±0.05	1.60±0.03	1.62±0.03
10	1/7	1.71±0.06	1.74±0.07	1.89±0.04	1.93±0.05	1.55±0.19	1.61±0.06
30	0/5	1.70±0.03	1.75±0.05	1.94±0.04	1.95±0.05	1.62±0.04	1.62±0.03
100	0/6	1.75±0.05	1.80±0.05	1.92±0.03	1.95±0.02	1.61±0.05	1.65±0.04
300	0/7	1.74±0.06	1.75±0.04	1.95±0.03	1.90±0.06	1.67±0.03	1.66±0.02
1000	0/5	1.67±0.08	1.74±0.08	1.88±0.06	1.94±0.04	1.61±0.07	1.62±0.07
*Male*							
0	0/7	1.73±0.02	1.75±0.03	1.92±0.02	1.95±0.02	1.61±0.03	1.61±0.01
10	0/7	1.75±0.03	1.80±0.02	1.96±0.04	1.97±0.02	1.65±0.02	1.63±0.02
30	0/9	1.74±0.02	1.79±0.03	1.93±0.02	1.97±0.02	1.63±0.02	1.65±0.02
100	0/7	1.78±0.02	1.77±0.03	1.95±0.02	1.97±0.02	1.65±0.02	1.67±0.01
300	0/6	1.79±0.03	1.79±0.03	2.00±0.05	2.00±0.04	**1.71±0.03**^a^	**1.69±0.03**^a^
1000	0/7	1.73±0.03	1.74±0.05	1.96±0.05	1.95±0.02	1.65±0.03	1.68±0.04

3MA = third molar agenesis; M_1_ = first mandibular molar; M_2_ = second maxillary molar; M_3_ = third maxillary molar

Statistically significant mean differences from the control group are bold.

^a^ *p*<0.05 vs. control

**Table 7:** Results of benchmark dose modeling showing significant PCB 180 dose-responses of tooth size (maximum buccolingual diameter) for all molars (A) and of tibial length, cortical bone mineral density, and thickness of diaphysis (B) on PND 84. Critical effect doses (mg/kg bw) and their lower and upper bounds of the 95% confidence intervals are shown for critical effect sizes of 5% and 50%.

					CES 5% (mg PCB 180 / kg bw)			CES 50% (mg PCB 180 / kg bw)		
Endpoint	Side	Sex	Model	Doses (mg/kg bw)	CED	CED-L	CED-U	CED	CED-L	CED-U
*A*. *Buccolingual diameter of molars*										
M_1_	L	F	E2	0–1000	3300	1456	inf.	45000	19580	inf.
M^1^	L	M	E3	0–300	3391	0	inf.	n/a	n/a	n/a
	R	M	E5	0–300	1417	0.12	inf.	n/a	n/a	n/a
M_2_	L	F	E2	0–1000	2040	1178	7085	27600	15920	95750
	R	F	E2	0–1000	3470	0	inf.	46900	23260	inf.
M^2^	L	M	E2	0–300	489	286	1686	4061	2375	14013
	R	M	E3	0–300	329	0	inf.	n/a	n/a	n/a
M_3_	L	M	E2	0–300	247	170	453	2054	1413	3761
	R	M	E5	0–1000	455	3.71	1.2x10^7^	2x10^10^	6x10^5^	inf.
M^3^	L	F	E2	0–1000	725	536	1119	9793	7243	15117
		M	E2	0–300	441	248	1957	3663	2064	16264
	R	F	E2	0–1000	1091	606	5524	14753	8187	74647
		M	E2	0–300	559	n/a	n/a	4975	n/a	n/a
*B*. *Tibial length*, *cortical bone mineral density*, *and thickness of diaphysis*										
Length	R	M	E2	0–300	407	268	850	3385	2226	7066
BMD	R	M	E2	0–300	1002	702	1748	13538	9489	23625
Thick.	R	F	E2	0–1000	845	553	1795	7023	4593	14946
		M	E3	0–300	4x10^5^	n/a	n/a	n/a	n/a	n/a

M^1^ = first maxillary molar; M^2^ = second maxillary molar; M^3^ = third maxillary molar; M_1_ = first mandibular molar; M_2_ = second maxillary molar; M_3_ = third maxillary molar; BMD = bone mineral density; thick. = thickness; CED = critical effect dose; CED-L = lower bound of the 95% confidence interval of the critical effect dose; CED-U = upper bound of the 95% confidence interval of the critical effect dose; CES = critical effect size

In the mandibular dentition, the only significant differences between control and treatment groups were size increases of the M_3_ in males at 100 and 300 mg/kg bw (*p* = 0.001–0.025) and in females at 300 mg/kg bw (*p* = 0.001) (Table 6). Benchmark dose modeling indicated that size of M_3_ was the most sensitive endpoint in males (CED at CES 5% 247 and 455 mg/kg bw) followed by the size of M^2^, M^3^, and M^1^ (Table 7A). In females, the size of M^3^ was the most sensitive alteration (725–1091 mg/kg bw) followed by the size of M^2^ and M^1^.

### Tibial metrics

Mean and SD values for tibial measurements are reported by sampling time point, sex and dose in Table 1 and S2 and S3 Tables, and the outcome of dose-response modeling in Table 7B. Effects observed on PND 35 were milder and nonsignificant compared to those on PND 84. Tibial length was most strongly affected in males on PND 84, showing dose-dependent increases of 1–2 mm at 10, 100, and 300 mg/kg bw (*p* = 0.030, 0.020, and 0.002 respectively). For females, this effect was only observed at 10 mg/kg bw (*p* = 0.007). BMD of cortical diaphysis was decreased in males at 100–1000 mg/kg bw on PND 84 in nearly dose-dependent manner. Nonsignificant decreases in BMD were also observed at 1000 mg/kg bw in females on PND 84 and in males and females on PND 35. For both females and males, BMD of trabecular metaphysis was not affected, and only females showed significant effects on tibial bone area and thickness with increases at 300 and 1000 mg/kg bw on PND 84 (Table 1). Circumference, total BMD values, and biomechanical properties of tibial shafts were not affected by the treatment (S2 and S3 Tables). Benchmark dose modeling indicated that increased tibial length was the most sensitive endpoint in males (CED at CES 5% 407 mg/kg bw) followed by decreased cortical BMD (Table 7B). In females, the only tibial endpoint showing a significant dose-response was increased thickness of cortical diaphysis (CED at CES 5% 845 mg/kg bw).

### Correlations between variables

The results of the correlation tests showed strong linear relationships for all molars with CS, as well as with each other, in both sexes (S4 and S5 Tables). While FA was significantly correlated with M_3_ size in females (*r =* -0.4914, *p* = 0.005), it was correlated with CS and several postcranial variables in males. Most of the tibial measurements were significantly correlated with each other in both sexes.

## Discussion

This study links PCB 180 exposure to numerous skeletal and dental effects previously associated with TCDD and DL compounds. In particular, rat teeth have been identified as highly sensitive to *in utero*/lactational TCDD exposure for a variety of endpoints, such as reduced cusp size and enamel thickness, hypomineralization, caries susceptibility, arrested root formation, and total failure of tooth development or eruption [25–28]. Similar effects have also been reported in experimental studies of rhesus macaques [29] and mice [30], as well as in wildlife studies of voles [31] and human epidemiological studies [32–35].

The present study shows that PCB 180 and TCDD share some effects on craniofacial shape, e.g., shortening of the face and/or shift in the palatal suture with *in utero*/lactational exposure to high doses [14, 15], although few significant changes were observed overall. More notably, the present study indicates that molar teeth are also targets of developmental toxicity of PCB 180, and that the responses to PCB 180 differ from those due to exposure to DL compounds. Firstly, there is a marked potency difference of almost six orders of magnitude between effective doses of DL compounds and PCB 180: effects of TCDD were observed at maternal dose level of 30 ng/kg bw [25] and those of PCB 180 at 10 mg/kg bw. There are also several qualitative differences in the responses. The maxillary molars in females and M^3^ in males showed reduced cusp size at the highest dose levels of PCB 180, yet the males at all other dose levels showed significant *increases* in cusp size in all three maxillary molars as well as the M_3_. Similarly, the mandibular molars in females showed slightly (but mainly non-significant) increased size at most dose levels. Benchmark dose modeling showed that the increased size of M_3_s in male rats was the most sensitive target of PCB 180, whereas in other molars the dose responses were weak or absent.

It is important to note that the molars of the rat do not grow continuously, unlike the incisors, and that enamel does not remodel or heal once formed, unlike bone tissue. During the gestation period of the rat, which normally lasts for about 21 days, the first, second, and third molars are initiated around GD 14, 15 and 21 and fully functional around PND 25, 28, and 40, respectively. Mineralization follows initiation by one week in the first and second molars and two weeks in the third molar, with tooth eruption about three weeks after mineralization begins [28, 36]. In this sequence, the mineralization of the third molar occurs during the first two weeks of life, which was the period of highest TCDD body burden in offspring due to lactational exposure in our previous study [15]. Although the dams in the present study only received doses of PCB 180 during pregnancy, their nursing offspring would have experienced PCB 180 exposure via the mother’s milk during weaning, given its estimated elimination half-life of 90 days in rats [37]. Thus, as the molars preserve a permanent record of the process of enamel formation over the entire tooth crown, their altered size reflects the developmental disturbance of *in utero*/lactational exposure to PCB 180 during this entire period. Those rats also showed zero frequencies of the interfrontal bone among all treatment groups, possibly related to the fusion of the anterior frontal suture at the same time [15].

Similar to the molars, several tibial effects observed in this study are shared with the DL PCBs. Increased cortical thickness and decreased trabecular area in females, for example, has also been reported for 3,3',4,4',5-pentachlorobiphenyl (PCB 126) [38]. However, the effects on tibial length are different: moderate doses of PCB 180 increased the tibial length, while TCDD dose-dependently decreased it [39]. Likewise, positive correlation between cortical area parameters and PCB concentration has previously been reported in otters [40]. Also, there are marked sex differences in tibial effects of DL and NDL compounds: whereas TCDD and other DL compounds have shown stronger adverse effects on bone and teeth in females, here the males show greater sensitivity to PCB 180 exposure in general. This sexual dimorphism is evident not only in the cranium and teeth, but also in the tibia, which increased in length for males at moderate dose levels and showed no significant effect on length in females. In addition, the tibial measurements here showed few correlations with the cranial or dental variables, whereas in our previous study, decreased cranial size from *in utero*/lactational and adult exposure to TCDD was correlated with decreased long bone length in females of three different strains, including the TCDD-resistant Han/Wistar rats [15]. These correlations in the three rat strains could not be explained as effects of hypophagia, due to a weak correlation between CS and body weight gain, and may be the result of strain-specific variations in the AHR-mediated interference with the nuclear receptor-signaling pathways that are involved in skeletal development and growth.

Sex differences in the toxicological effects observed after exposure to TCDD and other high-potency DL compounds on the one hand, and PCB 180 on the other hand, can be attributed to variations in the ability to modulate several different types of biological responses to chemical exposures. For example, marked sex-based differences are commonly observed in the induction of cytochrome P450 (CYP) enzymes, which play a major role in the metabolism of various types of chemicals[41]. Studies of rat and mouse models have established that the sexually dimorphic expression of *CYP* genes is regulated by sex-dependent temporal patterns in the pituitary release of growth hormone (GH), which imparts sex differences to long bone length and overall body growth rates [42]. While DL PCBs induce CYP 1 enzymes (i.e., CYP 1A1, 1A2, and 1B1) by activation of the AHR, it appears that NDL PCBs activate the constitutive androstane receptor (CAR) and the pregnane X receptor (PXR), nuclear transcription factors that regulate *CYP2B1* and *CYP3A1* gene expression [43, 44]. Previously PCB 180 has shown a phenobarbital type of xenobiotic metabolism consistent with the induction of CYP2B1, but a lack of the typical AHR-dependent responses, such as CYP1A1 induction, thymus atrophy and permanent body weight reduction [19, 45]. Recent research has shown that CAR in particular has a major role in NDL PCB-driven induction of CYPs [44] and can lead to the sexually dimorphic induction of the CYP2B1 gene in rats [46]. The greater prominence of CAR may explain the stronger hepatic effects in male rats with adult exposure to PCB 180 [45], as well as the skeletal and dental effects observed in the present study, due in part to the greater suppression of CYP2B induction by continuous GH secretion in females compared to episodic GH secretion in males [47]. In addition, *in vitro* toxicity profile data for PCB 180 have shown weak antiestrogenic potency but moderate antiandrogenic potency [48] as well as hypothyroidism, possibly due to the reduced transport of thyroid hormones by transthyretin (TTR) and/or the induction of UDP-glucuronosyl tranferases (UGTs), the enzymes responsible for elimination of thyroid hormones [19]. As hypothyroidism causes impaired bone formation and growth retardation [49], these factors may also contribute to the developmental skeletal effects observed in the present study.

In addition to the role of receptor-mediated and other specific mechanisms, it is also plausible that the skeletal and dental effects of PCB 180 observed at the highest dose level are secondary effects due to general toxicity. Decreased maternal and neonatal body weight development at 1000 mg/kg bw, although mild and temporary, took place on GDs 9–14 and during the first postnatal week, respectively, which are potentially important critical windows of sensitivity for both dental and skeletal development [26, 36, 39, 50, 51], and may therefore have contributed to the observed alterations. This explanation is also consistent with the reversal of increased cusp size of maxillary molars and increased tibial length, all of which were observed only at the highest dose level of PCB 180.

## Conclusion

By combining 3D cranial morphometric, cranial non-metric, and dental and tibial metric data, this study provides a comprehensive assessment of skeletal effects of PCB 180. We show that teeth are particularly reflective of *in utero*/lactational PCB 180 exposure, and that there is an increased male sensitivity in both dental and skeletal effects. These altered patterns of normal skeletal development are compatible with modulations of the specific signaling pathways, including several nuclear receptors, and metabolic enzymes, which are involved in mediating the toxic effects of PCB 180. Also, general toxic effects as indicated by decreased maternal and neonatal body weight development at the highest dose level may have contributed to the observed effects. More research is needed to elucidate and understand the observed effects of PCB 180 on skeletal and dental development and their broader implications for human and animal health.

## Supporting information

S1 Fig Cranial variables analyzed in this study. A) Anatomical landmarks measured for geometric morphometrics (top = anterior view, bottom = posterior view). B) Inter-frontal bone assessed for non-metric analysis (indicated by white arrow). See Sholts et al. (2015) for additional information.
(TIF)

S2 Fig Scores on the first and second principal components (PC1 and PC 2) derived from the morphometric shape variables for female rat crania. Markers are color coded by dose (mg PCB 180/kg bw).
(TIF)

S3 Fig Scores on the first and second principal components (PC1 and PC 2) derived from the morphometric shape variables for male rat crania. Markers are color coded by dose (mg PCB 180/kg bw).
(TIF)

S4 Fig Lollipop graph of the shape changes associated with the first principal component (PC1) derived from the morphometric shape variables for female rat crania (anterior left and posterior right). The lines represent the magnitude and direction of change from each landmark in the consensus configuration.
(TIF)

S5 Fig Lollipop graph of the shape changes associated with the second principal component (PC2) derived from the morphometric shape variables for female rat crania (anterior left and posterior right). The lines represent the magnitude and direction of change from each landmark in the consensus configuration.
(TIF)

S6 Fig Lollipop graph of the shape changes associated with the first principal component (PC1) derived from the morphometric shape variables for male rat crania (anterior left and posterior right). The lines represent the magnitude and direction of change from each landmark in the consensus configuration.
(TIF)

S7 Fig Lollipop graph of the shape changes associated with the second principal component (PC2) derived from the morphometric shape variables for male rat crania (anterior left and posterior right). The lines represent the magnitude and direction of change from each landmark in the consensus configuration.
(TIF)

S1 Table Definitions of landmarks used for morphometric analysis. (PDF)

S2 Table Tibial geometry and bone mineral density (BMD) measurements of male and female offspring by dose of PCB 180 and sampling time point (group mean ± SD). This table includes data that are not shown in Table 1.
(PDF)

S3 Table Biomechanics measurements of male and female offspring by dose of PCB 180 and sampling time point (group mean ± SD). (PDF)

S4 Table Correlation coefficients (r) (above the diagonal) and significance values (p) (below the diagonal, shaded) between cranial, dental, and postcranial variables among females. (PDF)

S5 Table Correlation coefficients (r) (above the diagonal) and significance values (p) between cranial, dental, and postcranial variables among males. (PDF)

S1 Dataset Raw data underlying the findings of this study. (XLSX)
