# Supplementary figures and images for: Skeletal and dental effects on rats following in utero/lactational exposure to the non-dioxin-like polychlorinated biphenyl PCB 180
Source: PLoS One. 2017 Sep 28;12(9):e0185241. doi: 10.1371/journal.pone.0185241 (PMC5619758; doi:10.1371/journal.pone.0185241)

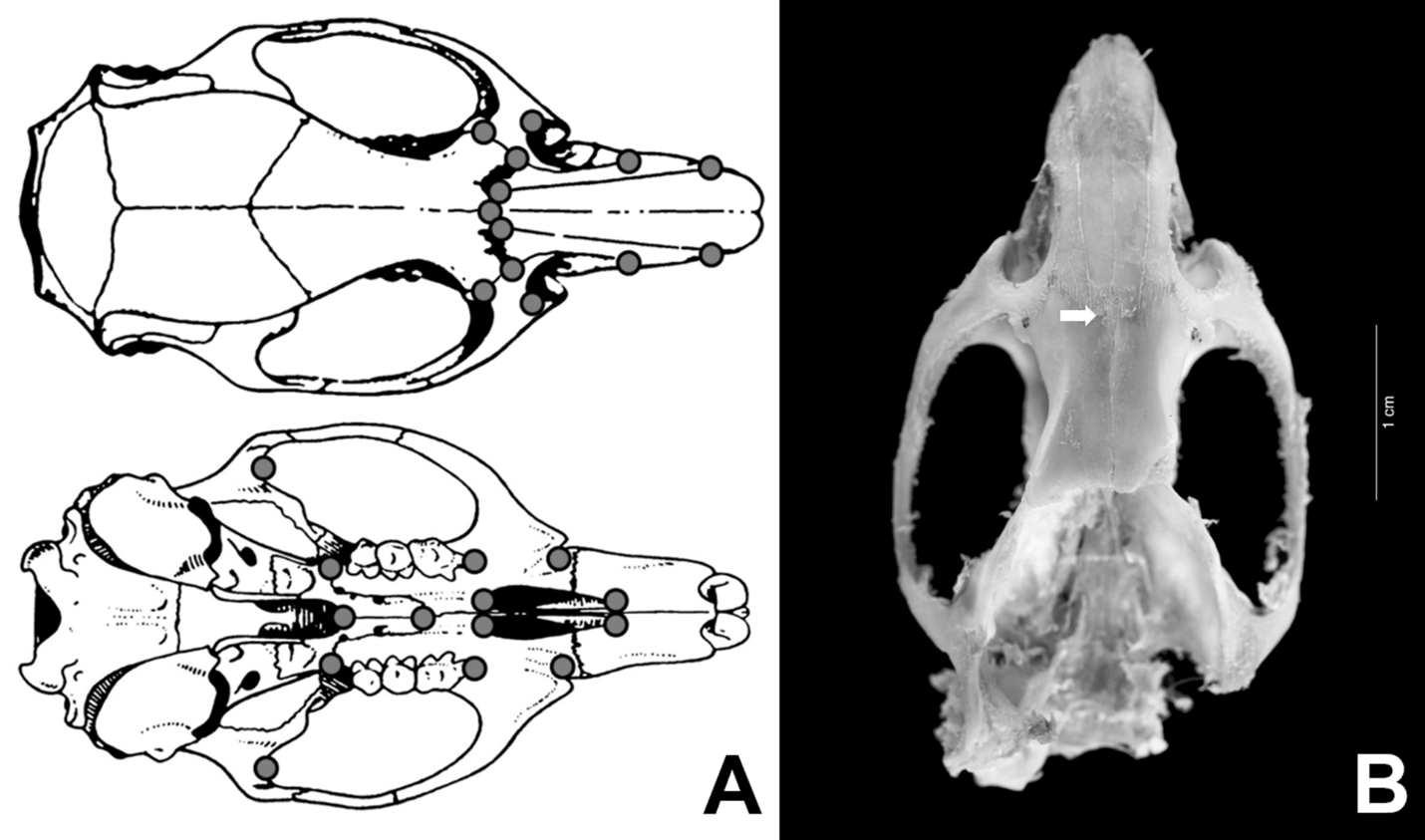

Supplement: S1 Fig — A) Anatomical landmarks measured for geometric morphometrics (top = anterior view, bottom = posterior view). B) Inter-frontal bone assessed for non-metric analysis (indicated by white arrow). See Sholts et al. (2015) for additional information. (TIF) [file pone.0185241.s001.tif]

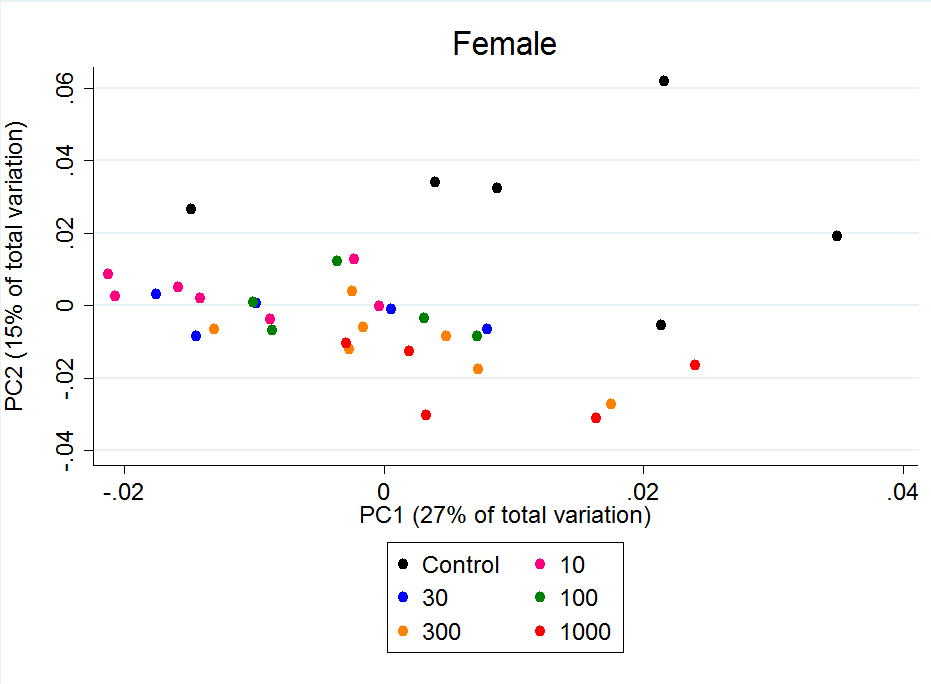

Supplement: S2 Fig — Markers are color coded by dose (mg PCB 180/kg bw). (TIF) [file pone.0185241.s002.tif]

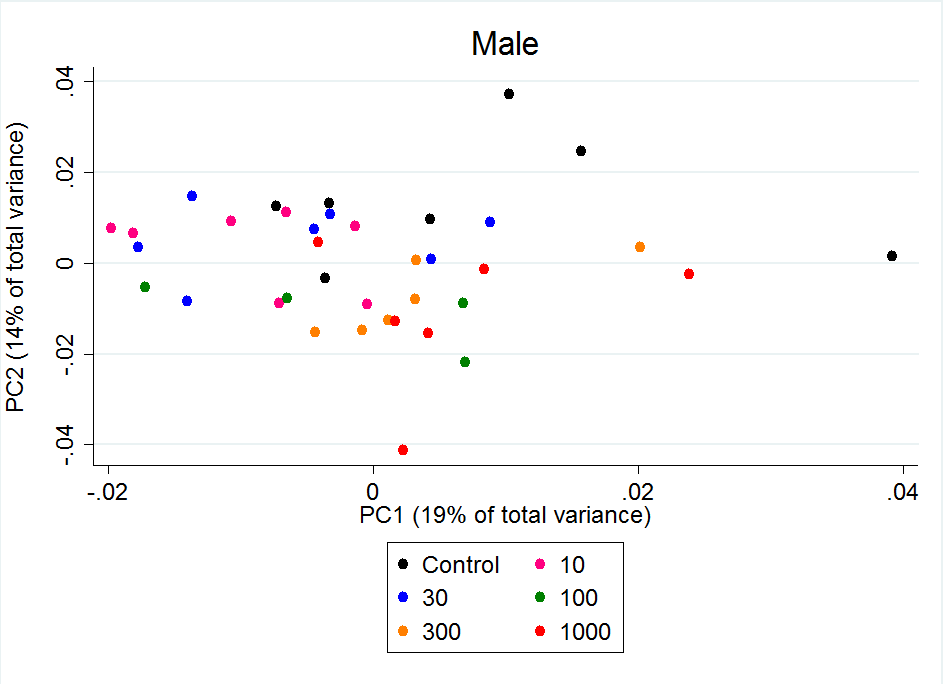

Supplement: S3 Fig — Markers are color coded by dose (mg PCB 180/kg bw). (TIF) [file pone.0185241.s003.tif]

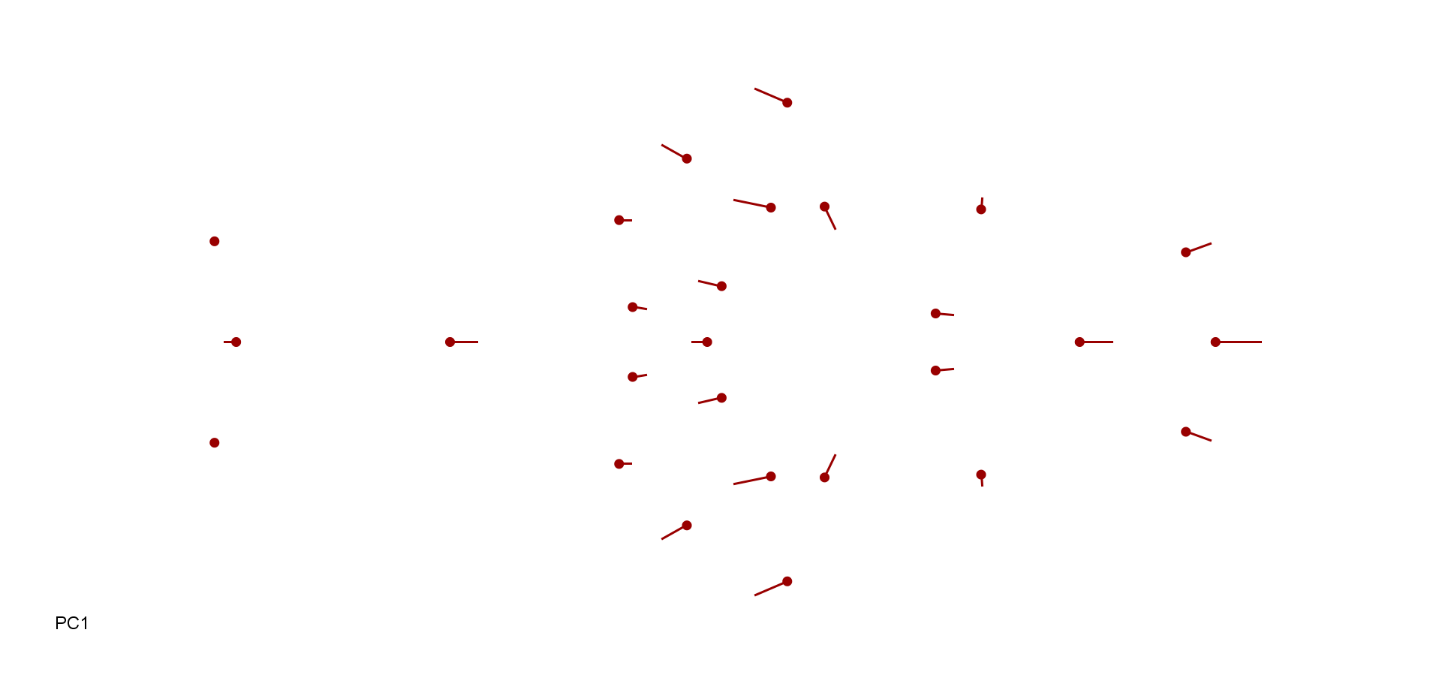

Supplement: S4 Fig — The lines represent the magnitude and direction of change from each landmark in the consensus configuration. (TIF) [file pone.0185241.s004.tif]

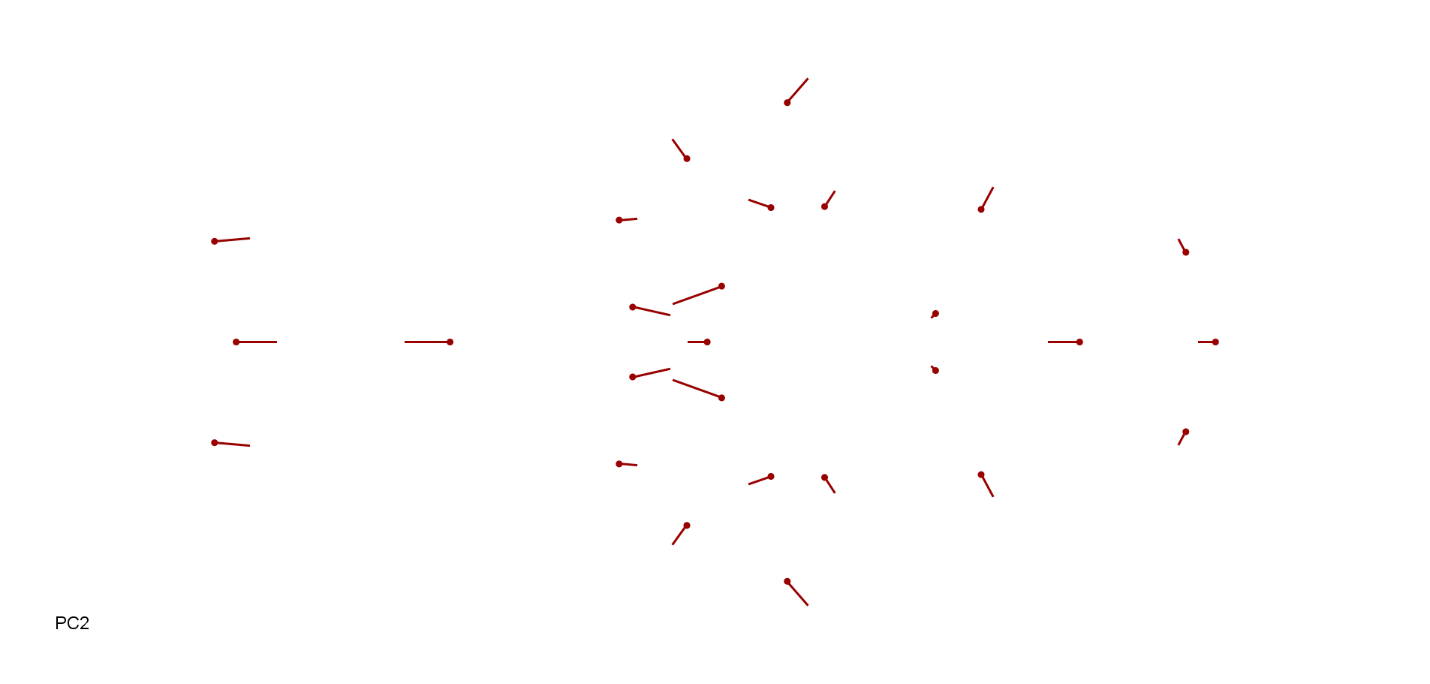

Supplement: S5 Fig — The lines represent the magnitude and direction of change from each landmark in the consensus configuration. (TIF) [file pone.0185241.s005.tif]

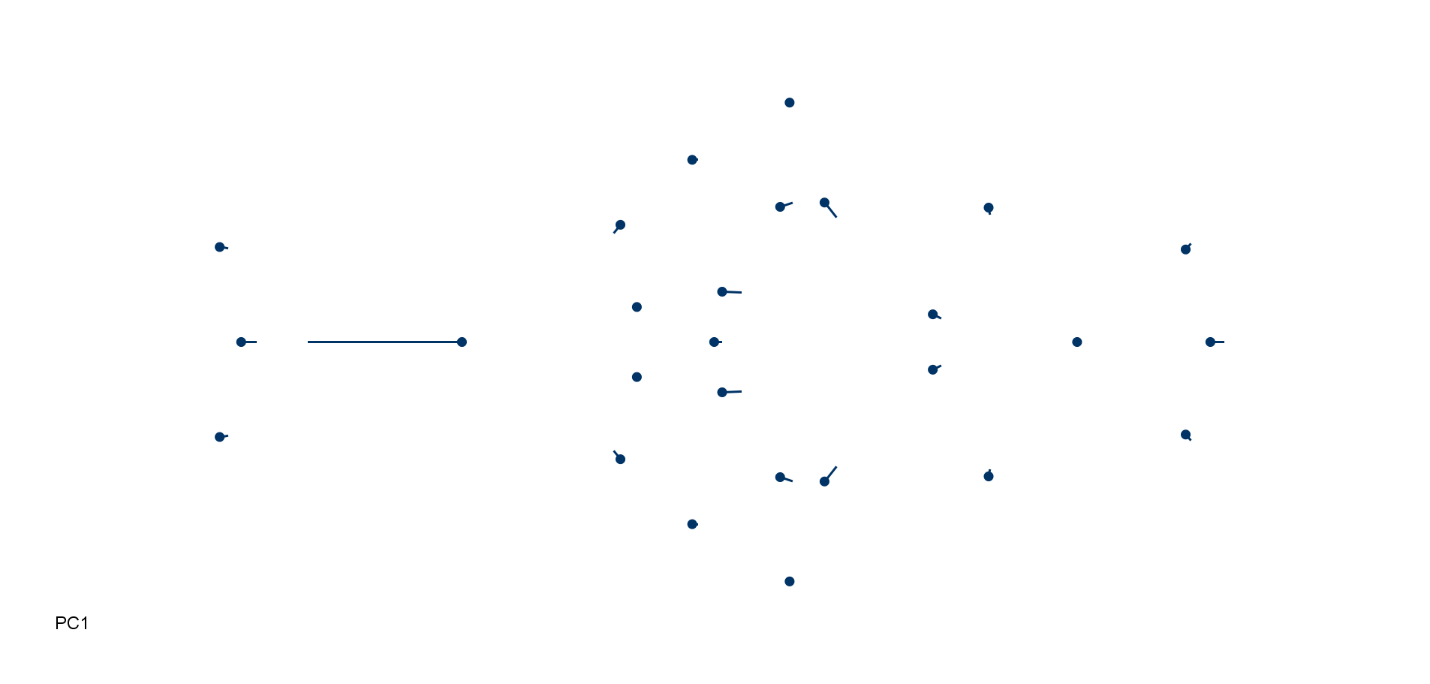

Supplement: S6 Fig — The lines represent the magnitude and direction of change from each landmark in the consensus configuration. (TIF) [file pone.0185241.s006.tif]

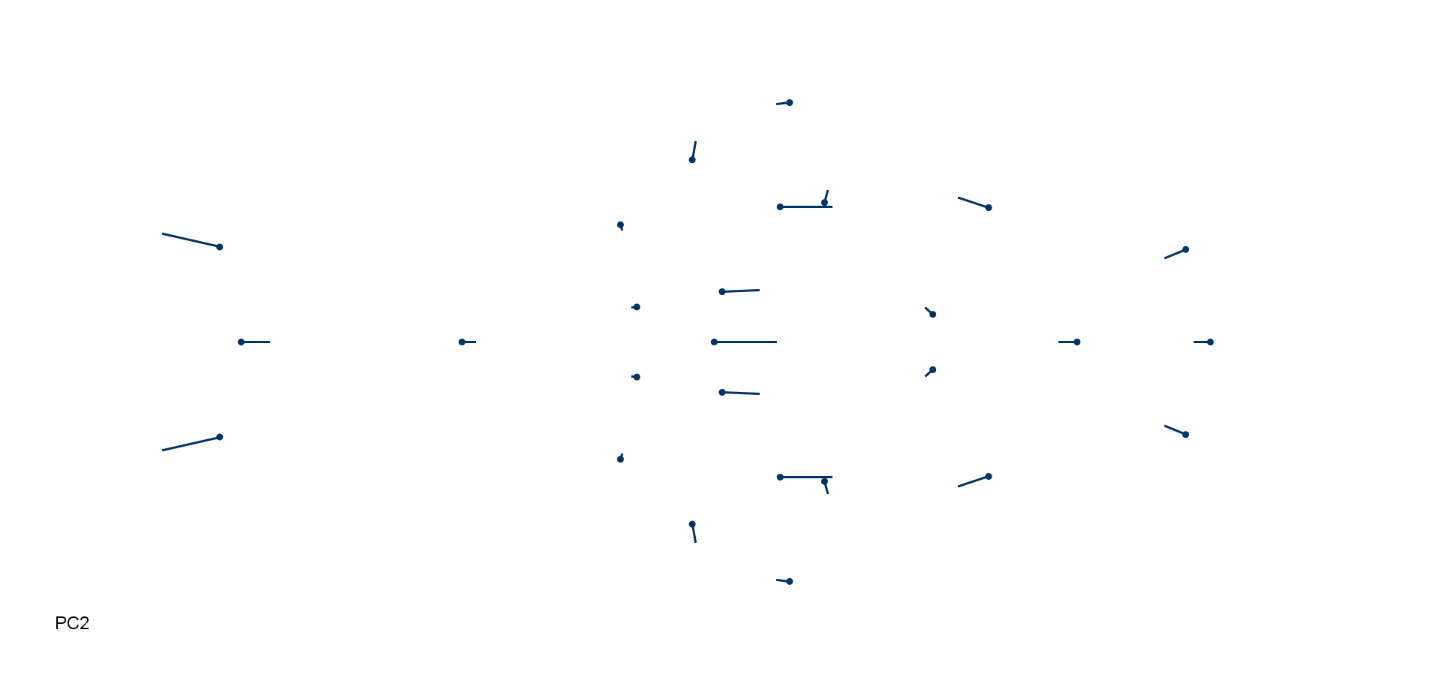

Supplement: S7 Fig — The lines represent the magnitude and direction of change from each landmark in the consensus configuration. (TIF) [file pone.0185241.s007.tif]
