# Supplementary material for: Skeletal and dental effects on rats following in utero/lactational exposure to the non-dioxin-like polychlorinated biphenyl PCB 180
Source: PLoS One. 2017 Sep 28;12(9):e0185241. doi: 10.1371/journal.pone.0185241 (PMC5619758; doi:10.1371/journal.pone.0185241)
Supplement: S1 Table — (PDF) [file pone.0185241.s008.pdf]

Paired	Module	Landmark Definition
X	Face	Anteriormost nasal – premaxilla intersection, left
X	Face	Anteriormost nasal – premaxilla intersection, right
X	Face	Anteriormost point on maxilla_left
X	Face	Anteriormost point on maxilla_right
X	Face	Point of deepest lateral incuravture on the superior aspect of the infra-orbital fissure, left
X	Face	Point of deepest lateral incuravture on the superior aspect of the infra-orbital fissure, right
X	Face	Frontal-premaxilla-maxilla intersection, left
X	Face	Frontal-premaxilla-maxilla intersection, right
X	Face	Frontal-premaxilla-nasal intersection, left
X	Face	Frontal-premaxilla-nasal intersection, right
	Face	Nasion (frontal-frontal-nasal-nasal intersection)
X	Face	Frontal-lacrimal-maxilla intersection, left
X	Face	Frontal-lacrimal-maxilla intersection, right
X	Face	Anteriormost premaxilla-premaxilla intersection between the incisors
X	Face	Inferiormost premaxilla-premaxilla intersection between the incisors
X	Face	Anteriormost margin of incisive foramen, right
X	Face	Anteriormost margin of incisive foramen, left
X	Face	Point of deepest posterior incuravture on the inferior aspect of the infra-orbital fissure, right
X	Face	Point of deepest posterior incuravture on the inferior aspect of the infra-orbital fissure, left
X	Face	Posteriormost margin of incisive foramen, right
X	Face	Posteriormost margin of incisive foramen, left
X	Face	Anteriormost point on the M1 at the alveolar bone, right
X	Face	Anteriormost point on the M1 at the alveolar bone, left
X	Base	Lateral palatal-pterygoid intersection, right
X	Base	Lateral palatal-pterygoid intersection, left
	Base	Posterior palatine-palatine intersection
	Base	Anterior palatine-palatine intersection
