# Supplementary material for: Skeletal and dental effects on rats following in utero/lactational exposure to the non-dioxin-like polychlorinated biphenyl PCB 180
Source: PLoS One. 2017 Sep 28;12(9):e0185241. doi: 10.1371/journal.pone.0185241 (PMC5619758; doi:10.1371/journal.pone.0185241)
Supplement: S2 Table — This table includes data that are not shown in Table 1. (PDF) [file pone.0185241.s009.pdf]

Dose (mg/kg bw)	N	Circumference, diaphysis		BMD, total	
		Periosteal (mm)	Endosteal (mm)	Diaphysis (mg/cm^3)	Metaphysis (mg/cm^3)
Female, PND 35					
0	7	6.43 ± 0.24	4.51 ± 0.20	718 ± 16	348 ± 32
10	7	6.47 ± 0.17	4.60 ± 0.14	701 ± 26	359 ± 11
30	7	6.60 ± 0.52	4.60 ± 0.36	718 ± 36	359 ± 36
100	7	6.37 ± 0.19	4.48 ± 0.14	715 ± 20	356 ± 16
300	7	6.58 ± 0.28	4.56 ± 0.21	725 ± 26	363 ± 32
1000	6	6.57 ± 0.15	4.59 ± 0.10	714 ± 19	339 ± 20
Male, PND 35					
0	7	6.56 ± 0.48	4.65 ± 0.29	704 ± 28	339 ± 28
10	7	6.61 ± 0.28	4.69 ± 0.17	693 ± 18	343 ± 18
30	7	6.85 ± 0.24	4.80 ± 0.14	716 ± 23	355 ± 11
100	7	6.60 ± 0.26	4.65 ± 0.18	713 ± 35	338 ± 16
300	7	6.79 ± 0.33	4.80 ± 0.19	697 ± 43	350 ± 25
1000	6	6.70 ± 0.22	4.71 ± 0.18	707 ± 25	347 ± 20
Female, PND 84					
0	7	8.51 ± 0.22	5.28 ± 0.23	912 ± 26	616 ± 11
10	7	8.52 ± 0.14	5.21 ± 0.23	930 ± 27	638 ± 19
30	5	8.56 ± 0.19	5.25 ± 0.16	918 ± 15	656 ± 23
100	7	8.43 ± 0.23	5.15 ± 0.29	927 ± 34	643 ± 16
300	7	8.66 ± 0.20	5.25 ± 0.21	931 ± 25	639 ± 30
1000	5	8.62 ± 0.18	5.19 ± 0.17	938 ± 15	655 ± 16
Male, PND 84					
0	7	9.16 ± 0.51	5.66 ± 0.29	915 ± 22	502 ± 16
10	7	9.53 ± 0.18	5.84 ± 0.22	917 ± 20	498 ± 13
30	9	9.27 ± 0.20	5.63 ± 0.18	927 ± 26	505 ± 16
100	7	9.49 ±0.20	5.86 ± 0.13	895 ± 11	492 ± 18
300	6	9.61 ±0.22	5.89 ± 0.18	906 ± 20	484 ± 16
1000	7	9.33 ± 0.28	5.73 ± 0.24	898 ± 24	488 ± 22

BMD=bone mineral density
