# Supplementary material for: Skeletal and dental effects on rats following in utero/lactational exposure to the non-dioxin-like polychlorinated biphenyl PCB 180
Source: PLoS One. 2017 Sep 28;12(9):e0185241. doi: 10.1371/journal.pone.0185241 (PMC5619758; doi:10.1371/journal.pone.0185241)
Supplement: S3 Table — (PDF) [file pone.0185241.s010.pdf]

Dose (mg/kg bw)	N	Biomechanics, diaphysis		
		Stiffness (N/mm)	Maximum Force (N)	Energy absorption (mJ)
Female, PND 35				
0	7	70.0 ± 11.7	31.8 ± 5.2	40.0 ± 18.4
10	7	71.9 ± 15.6	35.0 ± 3.4	32.9 ± 8.9
30	7	74.5 ± 12.7	33.8 ± 4.7	39.1 ± 23.3
100	7	75.3 ± 23.5	30.1 ± 3.7	26.3 ± 9.5
300	7	96.0 ± 19.3	34.7 ± 6.2	30.5 ± 5.0
1000	6	71.1 ± 11.5	32.0 ± 2.5	57.7 ± 31.5
Male, PND 35				
0	7	69.3 ± 10.0	31.3 ± 8.2	31.4 ± 23.4
10	7	74.6 ± 9.7	33.7 ± 4.9	26.4 ± 5.2
30	7	93.1 ± 19.9	34.1 ± 5.1	23.9 ± 4.9
100	7	73.1 ± 19.0	32.2 ± 6.4	31.0 ± 16.9
300	6	87.2 ± 43.4	33.1 ± 9.1	24.9 ± 9.7
1000	6	74.7 ± 9.6	32.5 ± 4.7	31.8 ± 22.7
Female, PND 84				
0	7	281.7 ± 27.8	123.5 ± 17.5	65.6 ± 15.1
10	6	279.3 ± 27.3	127.6 ± 19.7	85.1 ± 48.7
30	5	303.6 ± 43.8	118.7 ± 8.0	51.1 ± 15.3
100	7	245.5 ± 46.9	117.9 ± 3.3	93.4 ± 55.0
300	7	296.6 ± 63.5	129.2 ± 8.2	67.1 ± 10.5
1000	4	281.3 ± 52.6	122.6 ± 12.2	101.8 ± 70.9
Male, PND 84				
0	7	231.0 ± 25.2	143.5 ± 23.2	103.8 ± 21.3
10	7	239.0 ± 25.7	154.0 ± 14.6	93.6 ± 17.2
30	9	237.2 ± 60.9	140.4 ± 21.1	146.9 ± 129.3
100	7	224.9 ± 22.6	152.0 ± 14.6	86.7 ± 19.0
300	7	265.8 ± 29.2	146.8 ± 16.4	105.8 ± 7.5
1000	7	250.9 ± 37.5	153.1 ± 28.6	134.3 ± 34.2
