# Supplementary material for: Skeletal and dental effects on rats following in utero/lactational exposure to the non-dioxin-like polychlorinated biphenyl PCB 180
Source: PLoS One. 2017 Sep 28;12(9):e0185241. doi: 10.1371/journal.pone.0185241 (PMC5619758; doi:10.1371/journal.pone.0185241)
Supplement: S4 Table — (PDF) [file pone.0185241.s011.pdf]

| | $M^1$ | $M^2$ | $M^3$ | $M_1$ | $M_2$ | $M_3$ | CS | FA | length | cort_bmd | cort_a | cort_t | trab_bmd | trab_a |
|---|---|---|---|---|---|---|---|---|---|---|---|---|---|---|
| $M^1$ | - | **0.7087** | **0.6392** | **0.6439** | **0.5585** | **0.5842** | **0.467** | -0.2781 | 0.3029 | 0.1280 | 0.0646 | 0.0403 | 0.0625 | 0.0617 |
| $M^2$ | 0.0000 | - | **0.5116** | **0.4341** | **0.5629** | 0.2352 | 0.2843 | 0.0030 | 0.1401 | 0.1162 | -0.0159 | -0.0074 | -0.1293 | 0.0362 |
| $M^3$ | 0.0000 | 0.0012 | - | **0.4483** | **0.5458** | **0.3389** | **0.388** | -0.1246 | 0.2092 | 0.1575 | -0.1742 | -0.1989 | -0.0084 | 0.0265 |
| $M_1$ | 0.0000 | 0.0082 | 0.0061 | - | **0.5552** | **0.4418** | 0.2987 | -0.2042 | 0.2374 | 0.1689 | -0.0156 | 0.1711 | -0.1450 | 0.0408 |
| $M_2$ | 0.0004 | 0.0004 | 0.0006 | 0.0004 | - | **0.4501** | **0.4930** | -0.0621 | 0.1099 | 0.0619 | -0.2764 | -0.1885 | -0.2567 | -0.0600 |
| $M_3$ | 0.0003 | 0.1806 | 0.0499 | 0.0089 | 0.0076 | - | 0.3362 | **-0.4914** | 0.2994 | 0.0684 | 0.2669 | 0.3339 | 0.1493 | -0.0183 |
| CS | 0.0054 | 0.1032 | 0.0234 | 0.0913 | 0.0036 | 0.0644 | - | -0.3159 | -0.0948 | -0.0958 | **-0.3927** | **-0.3866** | -0.1266 | -0.0051 |
| FA | 0.1112 | 0.9866 | 0.4827 | 0.2543 | 0.7314 | 0.005 | 0.0688 | - | -0.2785 | -0.0554 | -0.3041 | -0.3029 | 0.0738 | 0.6784 |
| length | 0.0684 | 0.4082 | 0.2140 | 0.1633 | 0.5233 | 0.0854 | 0.5937 | 0.1107 | - | **0.5980** | **0.4211** | **0.417** | -0.0495 | 0.2759 |
| cort_bmd | 0.4504 | 0.4935 | 0.3517 | 0.3248 | 0.7198 | 0.7008 | 0.5899 | 0.7557 | 0.0001 | - | 0.2538 | 0.2885 | -0.1462 | 0.2201 |
| cort_a | 0.7042 | 0.9257 | 0.3025 | 0.9281 | 0.1027 | 0.1271 | 0.0216 | 0.0804 | 0.0094 | 0.1295 | - | **0.8127** | 0.2101 | 0.2229 |
| cort_t | 0.8129 | 0.9651 | 0.2379 | 0.3183 | 0.2709 | 0.0536 | 0.0239 | 0.0816 | 0.0102 | 0.0833 | 0.0000 | - | 0.0673 | 0.0055 |
| trab_bmd | 0.7135 | 0.4458 | 0.9605 | 0.3988 | 0.1308 | 0.3992 | 0.4756 | 0.6784 | 0.7709 | 0.3880 | 0.2119 | 0.6921 | - | **-0.4245** |
| trab_a | 0.7166 | 0.8317 | 0.8764 | 0.8132 | 0.7283 | 0.9182 | 0.9773 | 0.5507 | 0.0983 | 0.1906 | 0.1848 | 0.9742 | 0.0088 | - |

$M^1$=maxillary first molar, left; $M^2$= maxillary second molar, left; $M^3$= maxillary third molar, left, $M_1$=mandibular first molar, left; $M_3$=mandibular second molar, left; $M_3$=mandibular third molar, left CS=centroid size; FA= fluctuating asymmetry; length=tibia length, left; cort_bmd=cortical bone mass density of tibia, left; cort_a=cortical area of tibia, left; cort_t=cortical thickness of tibia, left; trab_bmd= trabecular bone mass density of tibia, left; trab_a= trabecular area of tibia, left

Statistically significant correlations ($p<0.05$) are bold.
