# Supplementary material for: Skeletal and dental effects on rats following in utero/lactational exposure to the non-dioxin-like polychlorinated biphenyl PCB 180
Source: PLoS One. 2017 Sep 28;12(9):e0185241. doi: 10.1371/journal.pone.0185241 (PMC5619758; doi:10.1371/journal.pone.0185241)
Supplement: S5 Table — (PDF) [file pone.0185241.s012.pdf]

	M^1	M^2	M^3	M_1	M_2	M_3	CS	FA	length	cort_bmd	cort_a	cort_t	trab_bmd	trab_a
M^1	-	**0.8761**	**0.6585**	**0.8318**	**0.7854**	**0.6815**	**0.4007**	-0.1427	0.2160	-0.0064	0.2199	0.1906	0.1746	0.1729
M^2	0.0000	-	**0.7568**	**0.7385**	**0.7316**	**0.6944**	**0.5966**	-0.2413	0.291	0.044	0.2179	0.1867	0.2051	0.1586
M^3	0.0000	0.0000	-	**0.6119**	**0.4807**	**0.5661**	**0.6282**	-0.2249	0.2580	0.1365	0.1867	0.1574	0.0333	0.1105
M_1	0.0000	0.0000	0.0000	-	**0.7482**	**0.6675**	0.3913	-0.189	0.2849	0.0220	0.2753	0.2334	0.149	0.2414
M_2	0.0000	0.0000	0.0013	0.0000	-	**0.6629**	0.2989	-0.1611	0.2365	0.0832	0.1698	0.1689	0.1379	0.1055
M_3	0.0000	0.0000	0.0001	0.0000	0.0000	-	0.3245	-0.1266	0.2103	-0.1588	0.0787	0.0533	0.1802	0.1572
CS	0.0127	0.0001	0.0000	0.0166	0.0766	0.0535	-	**-0.3836**	0.2040	0.0626	0.0497	0.0395	-0.1741	0.0794
FA	0.3926	0.1445	0.1745	0.2626	0.3478	0.462	0.0174	-	**-0.3858**	-0.1051	-0.2478	-0.1833	0.0273	**-0.4053**
length	0.1590	0.0553	0.0909	0.0641	0.1316	0.1812	0.2192	0.0167	-	0.0296	0.6978	**0.5538**	-0.1162	**0.6909**
cort_bmd	0.9669	0.7766	0.3769	0.8887	0.6002	0.3152	0.7089	0.5299	0.8486	-	0.1205	0.2337	-0.1266	-0.1005
cort_a	0.1516	0.1554	0.225	0.074	0.2823	0.6205	0.7668	0.1336	0.0000	0.436	-	**0.9137**	0.1783	0.7908
cort_t	0.2152	0.2249	0.3077	0.1321	0.2848	0.7376	0.8137	0.2706	0.0001	0.1268	0.0000	-	0.1936	0.5677
trab_bmd	0.257	0.1817	0.8302	0.3403	0.3840	0.2533	0.2959	0.8709	0.4525	0.4128	0.2470	0.2078	-	0.1445
trab_a	0.2618	0.304	0.4751	0.1188	0.5061	0.3200	0.6357	0.0116	0.0000	0.5163	0.0000	0.0001	0.3494	-

M^1=maxillary first molar, left; M^2= maxillary second molar, left; M^3= maxillary third molar, left, M_1=mandibular first molar, left; M_3=mandibular second molar, left; M_3=mandibular third molar, left CS=centroid size; FA= fluctuating asymmetry; length=tibia length, left; cort_bmd=cortical bone mass density of tibia, left; cort_a=cortical area of tibia, left; cort_t=cortical thickness of tibia, left; trab_bmd= trabecular bone mass density of tibia, left; trab_a= trabecular area of tibia, left

Statistically significant correlations (p<0.05) are bold.
